# Case Report: Rare pleural effusion—a case of B-lymphoblastic lymphoma presenting with pleural effusion and diagnostic challenges

**DOI:** 10.3389/fmed.2025.1619612

**Published:** 2025-12-17

**Authors:** Fang Lu, Wenkang Zong, Liheng Yang, Dongrui Zhang, Wei Jia, Yi Li

**Affiliations:** 1 Department of Respiratory and Critical Care Medicine, Tianjin Chest Hospital, Tianjin, China; 2 Chest Hospital, Tianjin University, Tianjin, China; 3 Department of Pathology, Tianjin Chest Hospital, Tianjin, China

**Keywords:** B-lymphoblastic lymphoma, pleural effusion, tuberculous pleurisy, adenosine deaminase, CT-guided percutaneous pleural biopsy

## Abstract

**Background:**

Lymphoblastic lymphoma is a rare type of malignant lymphoma. B-lymphoblastic lymphoma with pleural effusion as the initial manifestation is extremely rare; in this study, we report a specific and challenging diagnostic process.

**Case presentation:**

We present the case of a 67-year-old woman who was initially diagnosed with tuberculous pleurisy with a right pleural effusion. The pleural effusion was an exudate composed primarily of mononuclear cells with elevated adenosine deaminase (ADA) and lactate dehydrogenase (LDH). The cytological results of the pleural effusion were negative. The contrast-enhanced CT suggested right pleural nodules with partial pleural thickening. Subsequently, a CT-guided percutaneous needle pleural biopsy was performed, and pathological consultation, bone marrow biopsy, and flow cytology examination were carried out. Finally, B-lymphoblastic lymphoma was diagnosed. During a recent follow-up, the patient showed signs of improvement nearly without pleural effusion after chemotherapy.

**Conclusion:**

Malignant pleural effusion caused by lymphoma can be easily misdiagnosed as tuberculous pleurisy, as both conditions commonly show markedly elevated levels of ADA and LDH. Flow cytology examination of pleural fluid can be considered, and histological specimens should be actively obtained to clarify the pathological diagnosis. For patients with extensive pleural thickening, a CT-guided percutaneous pleural biopsy can be preferentially selected.

## 1 Introduction

Tuberculous and malignant pleural effusion (MPE) are two common causes of exudative pleural effusion. Lymphoma is the third most common cause of MPE (1). It often shows an increase in lymphocytes with elevated adenosine deaminase (ADA), which is usually confused with tuberculous pleurisy (2, 3). Lymphoblastic lymphoma (LBL) is a rare malignant lymphoma that accounts for 2–4% of non-Hodgkin lymphoma in adults and is common in young men aged 10–30 years (4). The incidence of LBL is approximately 1.3–1.46 per 100,000 persons per year (4). It starts insidiously and usually presents with symptoms in the late stages of the disease. The main difference between LBL and acute lymphoblastic leukemia is the 25% infiltration degree of bone marrow lymphoblasts. According to the immunophenotype, it can be divided into T-lymphoblastic lymphoma (T-LBL) and B-lymphoblastic lymphoma (B-LBL), of which T-LBL accounts for approximately 90% (5). There are differences in clinical characteristics between T-LBL and B-LBL. T-LBL mainly presents with mediastinal and bone marrow involvement, often with mediastinal masses and pleural effusion as initial symptoms (4), while B-LBL usually shows extensive extranodal invasion, such as in the skin, the bone, the jaw, and the neck (6). B-lymphoblastic lymphoma with pleural effusion as the initial manifestation is extremely rare. A retrospective study reported that among 49 lymphoma patients with pleural effusion as the main complaint, only 2 cases (4%) were diagnosed with B-LBL (7). A systematic search of CNKI and PubMed databases using the keywords “B-LBL” and “pleural effusion” revealed a near-absence of relevant case reports. Herein, we report the specific, diagnostically challenging process and follow-up of such a case. This article presents a case of B-LBL with the onset of pleural effusion. The biochemical and cytological results of pleural effusion obtained at the local hospital did not provide a clear conclusion; finally, CT-guided pleural biopsy confirmed the diagnosis, adding to the growing body of evidence on B-LBL diagnosis.

## 2 Case presentation

A 67-year-old woman farmer was referred to our hospital on 22 December 2024, with complaints of wheezing for 1 month, which had worsened over the past 10 days after activity. One month ago, she began experiencing post-activity dyspnea without any apparent cause, accompanied by cough and fatigue. There was no fever, hemoptysis, or weight loss. She had been admitted to a local hospital from 11 December 2024 to 17 December 2024, during which time a massive right-sided pleural effusion was identified. A complete blood count showed the following: white blood cells: 7.02 × 10^9^/L, neutrophil ratio: 76%, absolute lymphocyte value: 1.03 × 10^9^ /L, Hb: 12.8 g/dL, and platelets: 303 × 10^9^/L. The erythrocyte sedimentation rate was 35 mm/h, and C-reactive protein (CRP) was 11.25 mg/L. Subsequently, thoracocentesis was performed, yielding hemorrhagic, turbid fluid. Routine analysis of the pleural fluid revealed a specific gravity of 1.032 and a white blood cell count of 8.0 × 10^9^/L, with monocytes comprising 99% and polynuclear cells comprising 1%. Differential cell counts of the pleural fluid (sediment) revealed mesothelial cells 10%, neutrophils 20%, and lymphoid cells 70%; no eosinophils or epithelial cells were observed. Other analyses of the pleural effusion indicated the following: ADA: 48 U/L, LDH: 561 U/L, glucose: 5.29 mmol/L, protein: 30.6 g/L, and Cellular Keratin 19 Fragment (CYFRA21-1): 12.85 ng/mL. No tumor cells were detected. A CT scan showed multiple patchy high-density shadows in both lungs and a consolidation shadow in the middle lobe of the right lung, with enlarged mediastinal lymph nodes. The local hospital administered empirical anti-infective and symptomatic treatments, but the efficacy was suboptimal. Due to the persistent presence of pleural effusion, the patient visited our hospital for further diagnosis and treatment. She underwent a total hysterectomy for endometrial cancer 20 years ago, with a history of hypothyroidism for 15 years. She has no history of smoking or alcohol consumption and denies any relevant family history.

**Figure 1:**
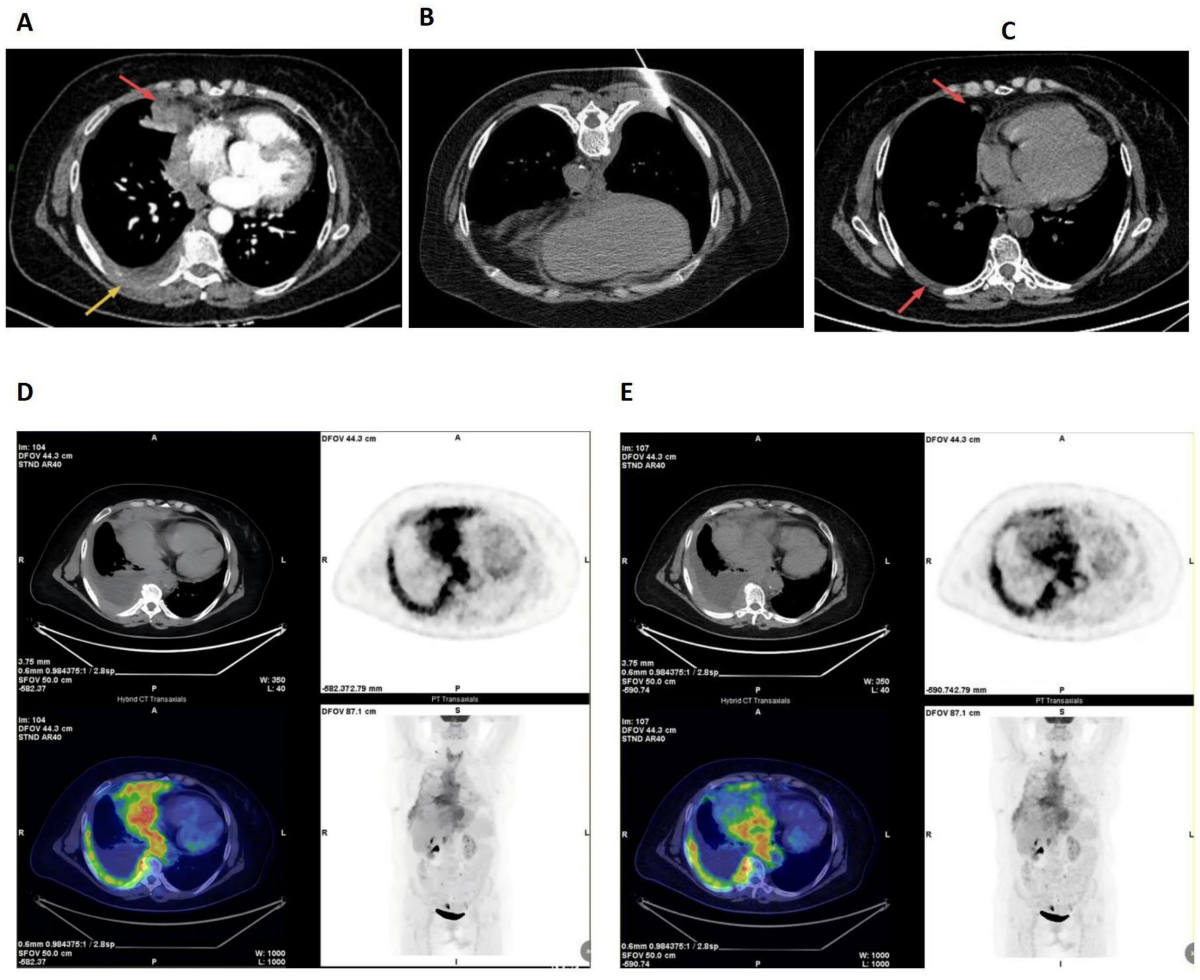
Relevant examination results. **(A)** Enhanced chest CT images on December 23, 2024: Irregular mass located nearby right lung(red arrow), and the right pleura shows thickening(yellow arrow). **(B)** CT-guided percutaneous pleura biopsy on December 24, 2024. **(C)** CT images after chemotherapy on February 3, 2025: Nodules and thickening of the right pleura were significantly reduced (red arrow). **(D,E)** Whole body fluorodeoxyglucose (FDG) positron emission tomography-computed tomography (PET-CT) (PET/CT) images on January 6, 2025: B-LBL involved the right pleura and mediastinum regions. **D** Chest and ediastinum involvement, **E** Sub-diaphragmatic involvement.

**Figure 2:**
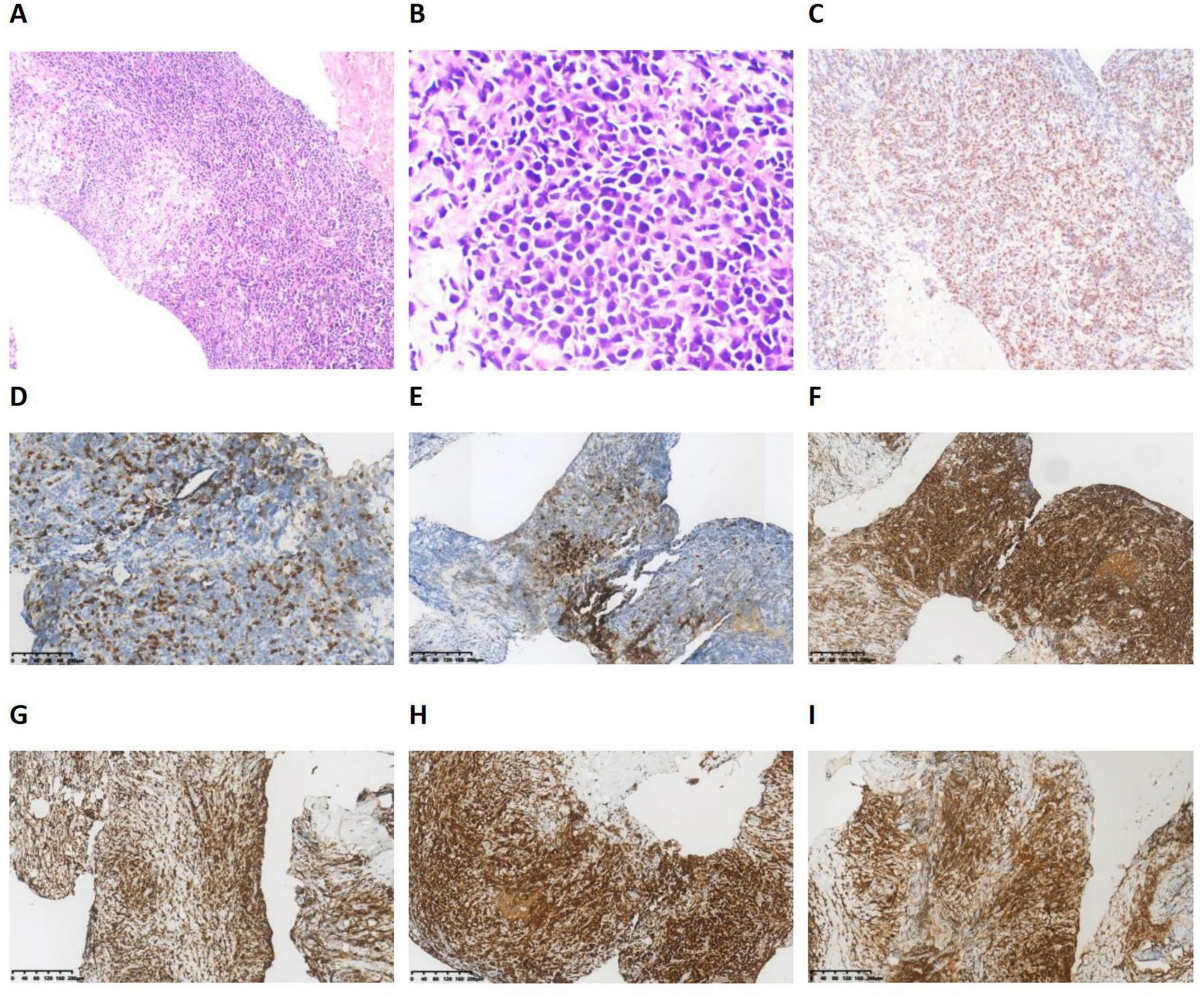
**(A–C)** The pleural biopsy showed a large number of deeply stained and squeezed atypical cells infiltration. **(A)** HE staining (Magnification *100). **(B)** HE staining (Magnification *400). **(C)** IHC staining shows a high expression of Ki-67. **(D–I)** The IHC staining: **(D)** CD3 (−), **(E)** CD20 (−). **(F)** CD43 (+), **(G)** CD79a (+). **(H)**PAX-5 (+), **(I)** TdT (+). HE, Hematoxylin–eosin; IHC, immunohistochemistry.

After admission, we initially administered piperacillin-tazobactam for infection control and provided expectorant therapy, while continuing drainage of the pleural effusion. Blood tests were normal, with a white blood cell count of 3.47 × 10^9^ /L, an absolute lymphocyte count of 1.06 × 10^9^ /L, and normal CRP at 5.8 mg/L. Serum LDH was elevated at 210 U/L. The characteristics of the pleural effusion were as follows: specific gravity: 1.032, monocyte: 99%, ADA: 102.3 U/L, glucose: 0.5 mmol/L, protein: 40.1 g/L, and LDH: 1217 U/L. Xpert *Mycobacterium tuberculosis*/rifampicin (Xpert MTB/RIF) detection was negative. The pathological results from the pleural effusion sediment showed that inflammatory cells exuded with increased lymphocytes and mesothelial cell proliferation; only a few atypical cells were observed. A chest-enhanced CT showed an irregular soft tissue density shadow under the pleura in the middle lobe of the right lung, accompanied by irregular thickening of the right pleura (Figure 1A). Multiple enlarged lymph nodes were observed in the mediastinum and the right hilum of the lung. The above findings are considered to be malignant lesions. Thus, a CT-guided percutaneous pleural biopsy was performed (Figure 1B). The results of the biopsy showed that heteromorphic lymphocytes can be observed, with the exception of lymphocytes and monocytes. Immunohistochemistry results were as follows: LCA (+), CK (P), Ki-67 (+ > 50%), p53 (individual+), p63 (individual+), TTF-1 (−), CD68 (minority+), and calretinin (CR) (−) (Figures 2A–C). Subsequently, the patient was referred to Tianjin Cancer Hospital for a pathological consultation. A malignant tumor of the lymphohematopoietic system was suspected. Combining the results of morphology and immunohistochemistry, it was confirmed to be a diagnosis of B lymphoblastic lymphoma (CD20(−), CD79a(+), CD3(−), TDT(+), CD34(+), CD43(+), PAX-5(+), CD19(+), CD1a(−), and MPO(−)) (Figures 2D–I).

**Figure 3:**
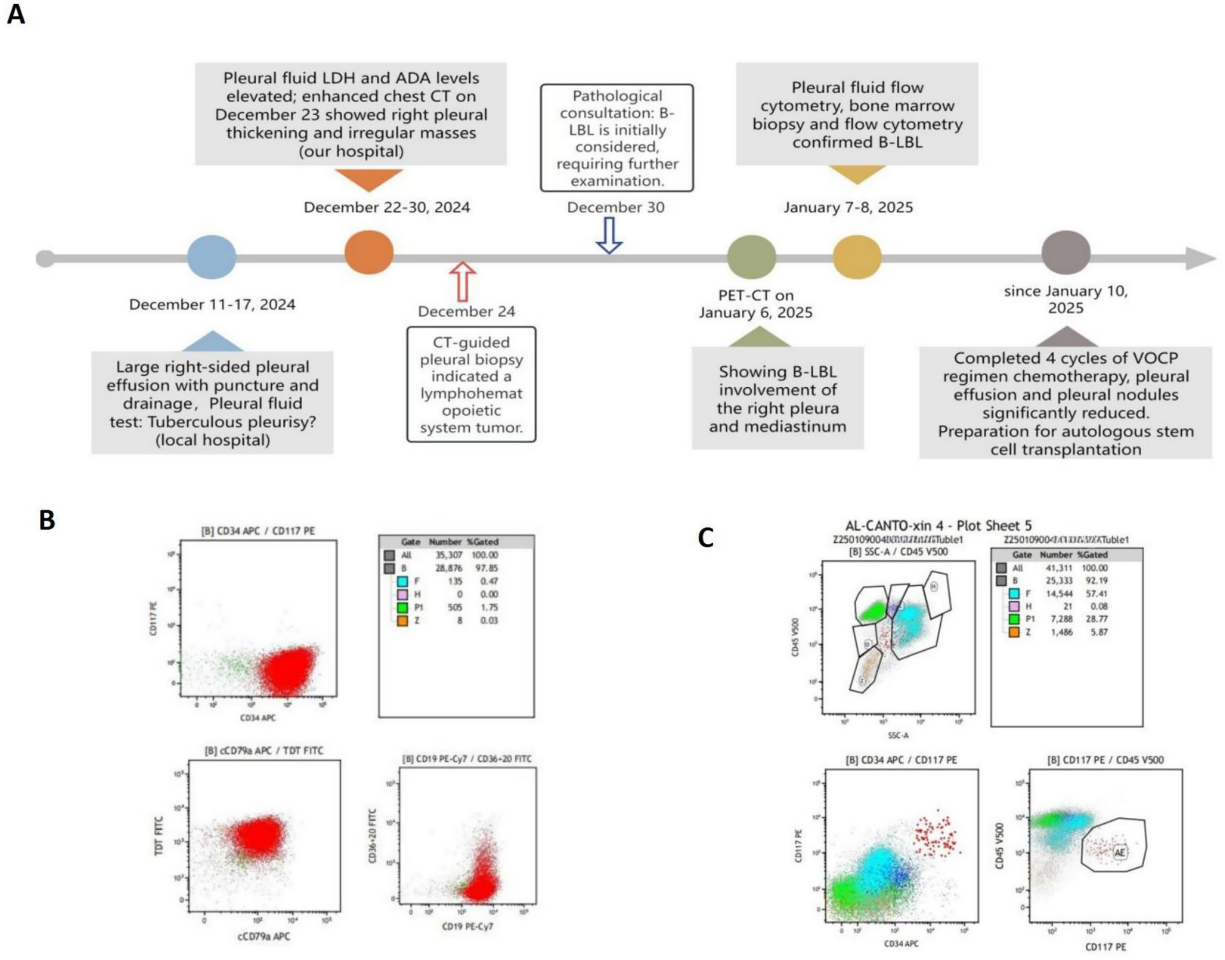
Timeline and Flow cytometry results. **(A)** Case Timeline **(B)** Flow cytometry of pleural effusion on January 7, 2025. **(C)** Flow cytometry of bone marrow on January 8, 2025.

The patient was referred to Tianjin Cancer Hospital, where flow cytometry of the pleural effusion was performed (Figures 3A,B). The results showed that there was a group of abnormal B lymphoblasts, accounting for 97.33% of the cells. These cells expressed CD34, CD38, HLA-DR, CD123, CD10, CD19, TDT, and CD22; partially expressed CD20; and weakly expressed cCD79a and CD13, consistent with the phenotype of acute B lymphoblastic leukemia (common B-ALL). Bone marrow aspiration, bone marrow biopsy, and immunophenotyping examinations were completed, which indicated trilineage hyperplasia without a significant increase in blast cells or abnormal lymphocytes (mature lymphocytes, 28.77%; myeloid blast cells, 0.37%) (Figure 3C). Subsequently, fluorodeoxyglucose (FDG) positron emission tomography-computed tomography (PET/CT) was performed, showing multiple nodules and masses in many areas of the chest and abdominal cavity, with no uptake in bone marrow (Figures 1D–E). There was a significant possibility of multi-system involvement by lymphoma, except for the bone marrow, as confirmed by a bone marrow biopsy. All of the results further supported the diagnosis of B-lymphoblastic lymphoma. The patient maintained an optimistic attitude and actively cooperated with the treatment. She has received four cycles of chemotherapy with the VOCP regimen since 10 January 2025 (vindesine 4 mg on days 1, 8, 15, 22, idarubicin 8 mg/m^2^ on days 1 and 15, cyclophosphamide 400 mg/m^2^ on days 1 and 15, prednisone 1 mg/kg/day from days 1 to 14, with a one-third dose reduction from days 15 to 28). During chemotherapy, severe leukopenia and thrombocytopenia developed, which improved following symptomatic management. A chest CT re-examination on 3 February 2025 revealed that the thickening of the right pleura and the nodules had decreased significantly (Figure 1C). The patient’s condition remains stable, and she is currently preparing for autologous stem cell transplantation.

## 3 Discussion

Pleural effusion caused by lymphoma is not easy to diagnose, and it is also prone to missed diagnosis or misdiagnosis (2). The mechanisms of pleural effusion caused by lymphoma vary. Obstruction of venous and lymphatic return caused by tumor compression plays a significant role among patients with Hodgkin lymphoma, which occurs in the later period of the disease and can manifest as transudate or chylothorax without tumor cells in pleural effusion (8). Approximately 20% of non-Hodgkin lymphomas can be accompanied by pleural effusion, which is primarily attributed to pleural metastasis (4, 9), and can occur in the early period. The characteristic of pleural effusion in non-Hodgkin lymphoma is exudative with increased lymphocytes and an elevated level of ADA, which is similar to tuberculous pleurisy. In a case report involving seven lymphoma patients with pleural effusion as the initial manifestation, four cases were once suspected of having tuberculous pleurisy and received anti-tuberculosis treatment (10).

ADA is an enzyme involved in purine metabolism and is an essential enzyme in the process of differentiation of pre-T cells into lymphocytes. An increase in ADA is mostly observed in tuberculous pleurisy and can also be noticed in lymphoma, leukemia, empyema, paragonimiasis, and rheumatoid arthritis (9). Previous studies have shown that ADA has the greatest diagnostic value when it is > 30–50 U/L. When ADA is > 40 U/L, the sensitivity and specificity for diagnosing tuberculous pleurisy are both > 80% (9, 11). In lymphoma patients with thoracic involvement, the ADA level in the pleural effusion is 67.4 U/L (range, 30.0–92.0 U/L) (12), whereas an ADA level of > 250 U/L is rarely noticed and occurs only in lymphoma or parapneumonic effusion (11). The ADA level in the pleural effusion of this patient was 48 U/L in the local hospital and 102 U/L in our hospital, which is consistent with the results of previous studies. There are two isoenzymes for ADA, ADA1 and ADA2. ADA1 is found in various lymphocytes and monocytes, and ADA2 is only found in monocytes. Therefore, ADA2 increases primarily in tuberculous pleurisy, while ADA1 increases primarily in lymphoma. Considering the mild increase in serum CRP, the elevated ADA in the pleural effusion, the thickening pleura, and the enlarged mediastinal lymph nodes without tumor cells in the pleural effusion, this patient was initially suspected of having tuberculous pleurisy, which made it difficult to distinguish from a malignant pleural disease.

LDH is a key enzyme in glycolysis that is responsible for the conversion of pyruvate into lactate and vice versa. It plays a crucial role in infection, inflammation, tumor progression, and metastasis. Kim et al. concluded that, when the cutoff value of serum LDH is higher than 460 U/L, the sensitivity of distinguishing between lymphoma-related pleural effusion and tuberculosis is 76%, and the specificity is 81% (3). The serum LDH level of the patient is between 202 and 210 U/L, which does not exceed 460 U/L. Although it is inconsistent with the previous study, the serum LDH level gradually decreased from the initial 210 U/L to 149 U/L after one cycle of chemotherapy. This dynamic change in LDH may reflect the treatment effect. Another study reported that the cutoff value of LDH for differentiating between malignant and non-malignant pleural effusion is 681.5 U/L (with a sensitivity of 75.8% and a specificity of 67.3%) (13). In addition, the ratio of serum LDH to pleural effusion ADA was used to distinguish between MPE and non-MPE. When the ratio was higher than 20, the sensitivity of diagnosing MPE was 95–98%, the specificity was 85–94% (14), but the efficiency for lymphoma decreased. The LDH in the pleural effusion of this patient was 561 U/L in the local hospital and 1,217 U/L in our hospital, which also suggests the possibility of MPE to a certain degree, but it has no direct diagnostic value for lymphoma. Therefore, the degree of increase in LDH and ADA in pleural effusion can help estimate the nature of the effusion before a pathological diagnosis is confirmed. We reviewed the data of five lymphoma patients with clinical features of pleural or pericardial effusion in our hospital in the past 5 years. We found that the level of ADA in pleural or pericardial effusion was higher than 40 U/L in two cases; serum LDH was elevated in two cases (both < 460 U/L); the ratio of serum LDH to ADA in pleural or pericardial effusion was higher than 20 in one case; and the LDH level in pleural effusion exceeded 681 U/L in three cases. (Table 1).

**Table 1:** Clinical data of lymphoma patients with pleural effusion/pericardial effusion as the initial clinical presentation.

Case	Sex	Age	Serum LDH	Mononucle ar cell	ADA	LDH	Protein	Glucose	Serum LDH/effusion	Serum LDH post-treatment
			(U/L)	Ratio (%)	(U/L)	(U/L)	(g/L)	(mmol/L)	ADA	(U/L)
Case 1 (pleural effusion)	Female	67	210	99	102.3	1,217	40.1	0.5	2.05	149
Case 2 (pleural effusion)	Male	71	195	95	9.9	131	26.5	5.25	19.70	-
Case 3 (pleural effusion)	Female	89	278	90	60	3,072	50.1	6.45	4.63	-
Case 4 (pericardial effusion)	Female	63	227	90	14.5	192	46.8	6.66	15.66	-
Case 5 (pleural effusion)	Male	79	346	60	9.6	3,725	36.1	1.17	36.04	-

ADA, adenosine deaminase; LDH, Lactate dehydrogenase.

Cytology has a limit in diagnosing lymphoma. Previous studies have reported that the positive rate is different depending on the tumor type, degree of differentiation, and the quality of the submitted specimens (2). Small mature lymphocytes could be observed in lymphoma with low malignancy, which are easily confused with reactive pleural effusion (11). The cytomorphology of lymphoblastic lymphoma varies greatly, and it can be manifested as small blast cells or large cells with light blue cytoplasm and prominent nucleoli. The malignant lymphocytes in serous cavity effusions are similar to those in the blood and invade lymph nodes, which have consistent immunophenotyping. Therefore, the flow cytometric analysis of effusions can improve the early diagnosis (15). In this case, the patient subsequently underwent a flow cytology examination of the pleural effusion, which confirmed the diagnosis of B-lymphoblastic lymphoma (B-LBL).

Histopathology is the gold standard for diagnosis and can further clarify the lymphoma subtype. Procedures such as medical thoracoscopy, percutaneous fine-needle biopsy of the pleura, CT-guided pleural biopsy, and mediastinoscopy should be considered to obtain histological specimens (16). Medical thoracoscopy (MT) is the gold standard for the diagnosis of exudative pleural effusion. Previous studies have demonstrated that the diagnostic positive rate of MT in cases of exudative pleural effusion with negative cytology exceeds 90% (17), and the positive rate for malignant pleural mesothelioma is 87.5% (9, 18). Another study showed that the positive diagnostic rate of MT was 90% for MPE induced by non-Hodgkin lymphoma (19). CT-guided pleural biopsy is also an effective alternative. Studies have demonstrated that its sensitivity is 40% greater than that of blind pleural biopsy (87% versus 47%) (20). In comparison with ultrasound-guided pleural biopsy, its sensitivity can be elevated by 25.7% (66.7% versus 82.4%, *p* = 0.029) (21). Moreover, for patients with a pleural thickness of ≥ 1 cm, the sensitivity of CT-guided pleural biopsy can be increased to 93.7% (21). In comparison to MT, the diagnostic sensitivity of CT-guided pleural biopsy for exudative pleural effusion for an unknown reason was 87.5 and 94.1%, respectively. There was no statistically significant difference between the two groups (22). Therefore, CT-guided percutaneous pleural biopsy can preferably be selected for patients with obvious pleural thickening or pleural nodules. CT images of the patient showed extensive pleural thickening, so a CT-guided pleural puncture biopsy was performed. Compared with medical thoracoscopy, it has less trauma, more accurate positioning, and can avoid bleeding complications (the enhanced chest CT examination of this patient suggested obvious enhanced signs in the locally thickened part of the right pleura), and satisfactory specimens can be obtained, which is also a good choice. Therefore, for patients with pleural effusion of unknown reason accompanied by pleural thickening, thoracoscopy or CT-guided pleural biopsy is a powerful means to improve the positive diagnostic rate.

This article also has certain limitations, as follows: (1) Flow cytometry of pleural effusion has high value in the diagnosis of hematopoietic system malignancies (7, 23, 24). When B-ALL/LBL is suspected, a combined detection of CD20, PAX5, and CD79a can be performed to improve the positive rate (7). In this case, when the pathology was initially suspected, early submission of pleural effusion for flow cytometry could enable early diagnosis; (2) No further lymphoma-related genetic testing was performed, which could have more precisely guided treatment decisions and optimized prognostic assessment.

In conclusion, we reported a rare case of B-lymphoblastic lymphoma presenting initially with pleural effusion and concluded the key points for differentiating it from tuberculous pleurisy. There should be a high degree of suspicion regarding the possibility of lymphoma in case of increased mononuclear cells with elevated ADA and LDH in exudative pleural effusion. In such instances, pleural fluid flow cytometry examination should be carried out. CT-guided needle pleural biopsy can be preferentially selected to obtain histological specimens from patients with extensive pleural thickening.

## Data Availability

The original contributions presented in the study are included in the article/supplementary material, further inquiries can be directed to the corresponding author.
